# Flexibility of exercise capacity during nestling feeding in blue tits

**DOI:** 10.1242/jeb.251043

**Published:** 2026-03-31

**Authors:** Elana Rae Engert, Andreas Nord, Fredrik Andreasson, Jan-Åke Nilsson

**Affiliations:** ^1^ Department of Biology, Section for Evolutionary Ecology, Lund University, Ecology building, Lund S-22362, Sweden; ^2^ Swedish Centre for Impacts of Climate Extremes (climes), Lund University, Lund 22362, Sweden

**Keywords:** Exercise capacity, Breeding birds, Parental effort, Ecophysiology

## Abstract

Nestling feeding in altricial birds is a physiologically demanding phase of the annual cycle. Accordingly, parental effort in breeding songbirds has been suggested to be limited by the maximum capacity to perform sustained aerobic work. However, the maximum capacity for exercise and flexibility thereof is poorly understood in free-living, breeding songbirds. We tested two predictions related to the flexibility of exercise capacity in breeding blue tits with increasing workload due to an increasing demand for nestling feeding as nestlings grow older and with experimentally increased brood size. We measured the maximum exercise-induced metabolic rate and endurance of parents both early on and at the peak of the nestling feeding period, and found that exercise capacity increases with nestling age. Parents of enlarged broods had higher exercise capacity than those with unmanipulated broods, but this was only true for young birds. We suggest that the exercise capacity of breeding birds is a phenotypically flexible trait and depends on the amount of exercise that has been performed in the preceding days. However, the potential for an increase in exercise capacity is likely to be affected by parental age and experience, behavior and resource availability, in addition to nestling age and brood size.

## Teaser

**Summary:** Exercise capacity is a phenotypically flexible trait in breeding blue tits that changes during nestling feeding and may depend on a combination of nestling demand, parental age and resource availability.

## INTRODUCTION

For a small songbird, the breeding season is an energetically demanding stage of the annual cycle (Macías-Torres et al., 2022; Merilä and Wiggins, 1997). Birds with altricial young feed their nestlings by short but frequent commutes to foraging patches at least until nestlings fledge from the nest. While foraging flights may only last a few seconds, cumulative flight distances can surpass 100 km in a single day of nestling feeding (Yu et al., 2024). The amount and quality of food delivered by the parents per nestling is of direct importance for fitness of the parents, because it influences the quality and viability of young when they become independent, which could affect their survival (Tinbergen and Boerlijst, 1990). Because of their energetically expensive mode of transportation, flight (Schmidt-Nielsen, 1972), and feeding requirements for successfully raising young, altricial birds could be particularly limited by their physical capacity for exercise during the breeding season.

The limits of parental effort have received much attention in the field of life-history and evolutionary theory. Lack (1947) hypothesized that clutch size is determined by the maximum number of nestlings that the parents can successfully feed. If breeding is well timed with prey phenology, food resources can be abundant and energy availability may not be the limiting factor (Williams, 2018). Instead, parental effort may be physiologically limited, as there are upper limits to aerobic work that birds must perform to feed nestlings, which may limit brood size. Drent and Daan (1980) proposed that birds have a maximum sustained metabolic rate of around 4 times basal metabolic rate (BMR) during nestling feeding, above which there is a risk of compromising survival and future breeding attempts.

One factor that is not often considered in discussions of the determinants of parental effort is the effect of training on exercise capacity. In humans and model mammals, training leads to a suite of physiological changes in the cardiovascular system that confer the ability to exercise at a higher intensity and for longer periods of time (for a review, see Hellsten and Nyberg, 2016). After a prolonged period of intense training in professional athletes, however, exercise capacity will stabilize at a maximum level (Płoszczyca et al., 2019). The physiological ceiling of aerobic capacity is determined by a combination of genetics, age, sex, nutritional and environmental factors in humans (Bouchard et al., 1999; Lundby and Robach, 2015), race horses (Walker et al., 2009) and mice (Downs et al., 2016).

Similar to humans, the maximum metabolic rate that can be achieved during exercise (MMR) is also a flexible trait in birds and is thought to be related to exercise endurance and sustained workload (McKechnie and Swanson, 2010). It has been shown that MMR increases with endurance training in swim-trained ducks (Butler and Turner, 1988), running-trained chickens (Brackenbury and El-Sayed, 1985) and flight-trained songbirds (Petit and Vézina, 2014; Zhang et al., 2015b). Prolonged flight training or experimentally increased work rate (i.e. feather clipping or modified feeders as described by Koetsier and Verhulst, 2011) has also been linked to increased flight muscle mass (Petit and Vézina, 2014; Zhang et al., 2015b, 2018), blood oxygen carrying capacity (Bury et al., 2019; Yap et al., 2021), muscle lipid oxidative capacity (Price et al., 2022), lipid transport and cellular metabolic intensity (Zhang et al., 2015a), and antioxidant defenses (Larcombe et al., 2010).

Under natural circumstances during periods of high energy expenditure, such as during migration or in winter, birds undergo biological changes similar to those seen with exercise training (Swanson, 2010). Before migration and at stopover sites, birds undergo preemptive changes to their behavior and body composition during the staging period (Butler, 1991). This includes hyperphagia and hypertrophy, meaning a dramatic increase in energy intake, fat stores and pectoralis muscle mass, changes in fuel use, BMR and oxidative capacity (Coulson et al., 2024; Jenni-Eiermann et al., 2002; Kvist and Lindstrom, 2003; Lindström et al., 1999). In terms of exercise capacity, Hahn et al. (2022) found that long-distance migrants had higher endurance and blood oxygen carrying capacity, but similar MMR, compared with medium distance migrants at a migration stopover site. Cold-acclimated birds also show an increase in pectoralis muscle mass, heart mass, cellular metabolic intensity, lipid transport and BMR (Swanson, 2010; Zhang et al., 2015a,b).

During the breeding season, it would be reasonable to expect that similar physiological adjustments are needed to accommodate an increased workload due to increased parental care as for migratory flight or winter thermogenesis. We predicted that free-living, breeding birds would show an increased exercise capacity due to a ‘training effect’ following an increased workload during chick rearing. To test our predictions, we measured the exercise capacity in a wild bird, the blue tit (*Cyanistes caeruleus*), early and towards the end of the asymptotic growth trajectory of their nestlings. During this period, the food requirements of the nestlings increase as their growth rate peaks (Kunz and Ekman, 2000), resulting in increased parental food provisioning rates (Barba et al., 2009). Additionally, to motivate some birds to work harder than others, we experimentally increased the brood size for some birds (García-Navas and Sanz, 2010; Nur, 1984). We investigated whether birds with an increased parental workload had a higher exercise capacity after a period of nestling feeding compared with the control group.

## MATERIALS AND METHODS

This study was carried out on *Cyanistes caeruleus* (Linnaeus 1758) during the breeding seasons of 2023 and 2024, in Southern Sweden in an area of roughly 70 square kilometers around Lake Krankesjön where around 500 nest boxes have been monitored each spring since 1983 (55°42′N, 13°28′E).

### Brood size manipulations

We monitored nests for signs of nest building starting in mid-April and checked nests every 7 days for eggs. We estimated laying date assuming that one egg is produced per day. We checked nests for hatchlings every afternoon, starting 11 days after the last egg was laid, which is one day before predicted hatching. We selected nests with 8–13 chicks to include in the experiments. On the 5th day after hatching (day 5), nestlings were either ringed or marked with nail polish on the toenails and weighed. On the same day, half of the experimental nests were enlarged by transferring 5 nestlings from a donor nest box to another nearby nest of the same nestling age. If the number of nestlings on day 5 differed from the original clutch size by more than 2, additional nestlings were transferred from the donor nest to make up the difference. Donor nests were not considered further in this study because reducing brood size can have unpredictable effects on the behavioral response of parents (Nilsson and Nord, 2017, 2018) and to allow for a higher sample size of control and enlarged broods. The other half of experimental nests were considered control nests, where nestlings were ringed and weighed in the same way as in enlarged nests. On day 14, we measured mass and tarsus and wing length of the nestlings, and ringed those that were not ringed on day 5.

We used 50 nests in 2023 and 43 nests in 2024, but because of some nests being abandoned or predated, or because of equipment malfunction, 40 nests in 2023 and 35 nests in 2024 were retained for the experiments (Table 1). Nests in each brood size category did not differ in brood size prior to manipulation or hatch date (brood size: *t*-test, *t*_73_=−0.76, *P*=0.45; hatch date: *t*_73_=0.034, *P*=0.97). After brood size manipulations, control nests had 8–13 nestlings (mean±s.d.: 10.2±1.4) and enlarged nests had 13–18 nestlings (15.4±1.4), which is within the natural brood size variation in the study population. Mean (±s.d.) nestling mass in control nests was higher than that in enlarged nests on day 5, post-manipulation (*t*-test: *t*_73_=2.40, *P*=0.019; control: 5.19±0.48 g, enlarged: 4.93±0.45 g).

**Table 1.** Sample sizes of male blue tits with unmanipulated (control) or experimentally enlarged broods and aged as being in their 2nd calendar year (2CY) or their 3rd or greater calendar year (3CY+)

Year	Brood size	*n*	Age	*n*
2023	Control	21	2CY	14
			3CY+	7
	Enlarged	19	2CY	14
			3CY+	5
2024	Control	18	2CY	8
			3CY+	10
	Enlarged	17	2CY	7
			3CY+	10

We only included male parental birds in this study. Males were aged as in their 2nd calendar year (2CY, i.e. first breeding season) or 3rd or greater calendar year (3CY+, i.e. 2nd or later breeding season) based on plumage characteristics. Nests of 2CY birds and 3CY+ birds had a similar hatch date (*t*_73_=1.73, *P*=0.09) and brood size prior to manipulation (*t*-test, *t*_73_=−0.12, *P*=0.91). In 2023, 5 enlarged and 5 control males were fitted with an accelerometer developed by the Lund Electronics Lab (Department of Biology, Lund University, Sweden) weighing 0.47 g with a leg-loop harness between days 12 and 13 as a part of a pilot study. The accelerometer weighed 4–5% of the birds' body mass (9.8–12.8 g) and did not affect body mass or any of the metabolic traits (all *P*>0.05). In 2024, all male birds in the study were fitted with a PIT tag taped to two color rings on one leg on day 5 after the first metabolic measurements. The pit tag together with the tape weighed approximately 0.3 g, i.e. 2–3% of the birds' body mass. On day 12, an antenna was placed just inside the hole of the nestbox to measure feeding frequency on day 13 and was removed when the bird was recaptured on day 14. Feeding frequency data were averaged over 11 h (06:00–17:00 h) on day 13 for each bird.

### Metabolic measurements

Birds were caught and transported by car for no more than 15 min to a lab for metabolic measurements. In both years, males were measured on day 14. In 2024, we also measured males on day 5 before brood size manipulation, to record a baseline for birds before the brood size manipulation, so these individuals were measured twice. One control male that could not be caught on day 14 was measured on day 15 instead. Measurements were conducted between 08:00 h and 18:00 h and the temperature inside the lab was similar to the outside temperature, which fluctuated between 20.7 and 24.9°C (mean±s.d.: 23.1±0.78°C). The mean (±s.d.) holding time between capture and measurement was 43±23 min (range: 13–122 min) in 2023 and 29±17 min (range: 10–85 min) in 2024.

We used a hop-flutter wheel (Chappell et al., 1999; Swanson et al., 2012; Wiersma et al., 2007; Zhang et al., 2015b) made from a hermetically sealed clear Plexiglas cylinder (25 cm diameter×15 cm wide, 7.4 l volume) with an inlet and outlet on either side at the point of rotation. The wheel was lined with a sheet of 2 mm thick rubber cork so that birds would get traction in the wheel. The wheel was controlled by a variable speed motor with a range of 0.1 to 1.1 m s^−1^. O_2_, CO_2_, subsample flow rate, barometric pressure (BP) and water vapor pressure (WVP) were measured at 1 Hz with an FMS (Field Metabolic System, Sable Systems, Las Vegas, NV, USA). The CO_2_ sensor was zero and span calibrated using reference gases (100% N_2_ and 5% CO_2_, 95% N_2_) and the WVP sensor was zero calibrated using Drierite (Hammond Drierite Company, Xenia, OH, USA) before the experiment began in each year. The O_2_ sensor was span calibrated using dry air every morning before the tests began. Air was pushed into the wheel at a rate of 3034.3±285.3 ml min^−1^ STPD (mean±s.d.). We used dry atmospheric air from a cannister of pressurized air for experiments in 2023. In 2024, we used an air pump to push outdoor air through the Drierite. The flow rate of incurrent dry air was measured using an M Series mass flow meter (Alicat Scientific, Tucson, AZ, USA) upstream of the metabolic chamber. Excurrent air was subsampled at 300 ml min^−1^ of the main flow rate using an external subsample pump in 2023 and the FMS internal subsample pump in 2024. Baseline air was recorded for at least 5 min before and after metabolic measurements of each bird.

At the start of the measurements, the bird was placed into the hop-flutter wheel covered with a white sheet for an acclimation period of 15 min, during which we measured baseline air for 5 min and pre-exercise resting metabolic rate (RMR) for 10 min. We then removed the sheet and started the wheel. The wheel started to spin at 0.4 m s^−1^ for 3 min, after which the speed was increased by 0.1 m s^−1^ every 3 min until the maximum speed of 1.1 m s^−1^ was reached. We stopped the wheel when the bird began to struggle to keep its place in the wheel and stopped fluttering. The duration of the test was recorded as endurance. All birds showed signs of exhaustion after the test, such as resting their belly on the floor, closing their eyes and panting. We left the bird inside the wheel for 15 min to measure post-exercise RMR. Afterwards, we released the bird back at its nest box.

Fractional concentrations of O_2_ and CO_2_ were corrected for water vapor dilution using Eqn 1 (from equation 8.7 in Lighton, 2018), where *X* is the gas concentration and *X*_dry_ is the corrected value:
(1)

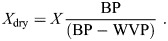
We calculated the rate of O_2_ consumption and CO_2_ production (*V̇*_O_2__ and *V̇*_CO_2__) using Eqns 2 and 3, respectively (equations 10.5 and 10.6 in Lighton, 2018):
(2)

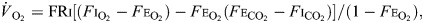

(3)

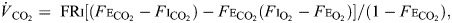
where FRi is flow rate into the chamber, *F*i_O_2__ and *F*e_O_2__ are the fractional concentrations of inspired and expired O_2_, and *F*i_CO_2__ and *F*e_CO_2__ are the fractional concentrations of inspired and expired CO_2_.

**Fig. 1.**
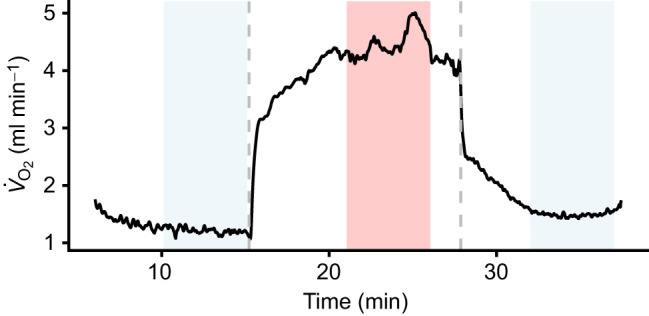
**Representative figure showing a recording of oxygen consumption (*V̇*_O_2__) during a test of exercise capacity of male blue tits.** Five minute selections were made based on the minimum rolling mean during a 15 min period of acclimation and post-exercise metabolic rate (blue shaded areas). The red shaded area shows a selection of the maximum metabolic rate (MMR), based on the 5 min maximum rolling mean during exercise (between the dashed lines).

We extracted RMR as the mean rolling 5 min minimum *V̇*_O_2__ from the initial acclimation period and post-exercise period and MMR as the mean rolling 5 min maximum *V̇*_O_2_ _during the exercise period (Fig. 1; Fig. S2). Oxygen consumption (*V̇*_O_2__, ml min^−1^) was converted to the energy equivalent in watts (W, J s^−1^), assuming an energy equivalence of 20 J ml^−1^ O_2_ (Kleiber, 1961). We defined endurance as the duration of the exercise period in minutes.

### Data analysis

We used R for statistical analyses (http://www.R-project.org/). Assumptions of all models were validated by visually inspecting residual plots. Endurance was log-transformed to fulfil the model assumptions. Estimated marginal means and pairwise comparisons of categorical variables were obtained using the *emmeans* package (https://CRAN.R-project.org/package=emmeans). All means and standard errors presented in the results are estimated marginal means (EMMs) derived from the models.

We evaluated the effect of brood size manipulations on the change in MMR, endurance and RMR within individuals between days 5 and 14 in 2024. We used linear mixed models (LMM) with body mass, MMR, endurance and RMR (pre- and post-exercise) as the dependent variables, brood size manipulation (control or enlarged) and day (5 or 14) as factors, time between when the bird was captured and the start of measurements (holding time) as a covariate and nest ID as a random intercept. Body mass was included as a covariate in all models except where it was the dependent variable. Age was not included in this model because of an insufficient sample size in each age–brood size category. We tested whether the difference between measurements on day 5 and day 14 depended on the brood size manipulation (day×brood size interaction).

To test for differences between brood size categories on day 14, 2023 and 2024 data were pooled and MMR, endurance and RMR (pre- and post-exercise) were used as dependent variables in linear models (LM). We included brood size manipulation (control or enlarged), age (2CY or 3CY+) and year (2023 or 2024) as factors and holding time and body mass as a co-variate in each model. We tested whether the effect of the brood size manipulation differed depending on age (age×brood size interaction).

To investigate the effects of the brood size manipulation, we modeled nest means of nestling body mass, wing length and tarsus length on day 14 as dependent variables in LMs. Brood size category, parent age and year were included as factors and hatch date was included as a covariate. Initial nestling mass (day 5) was included as a covariate in the model of nestling body mass. We tested whether the effect of brood size manipulations depended on male parent age (age×brood size interaction).

All experimental protocols comply with national legislation and were approved by the Malmö/Lund Animal Care committee prior to the start of the experiment (permit no. 5.8.18-07564/2022).

## RESULTS

### Change in exercise capacity before and after a brood size manipulation

**Fig. 2.**
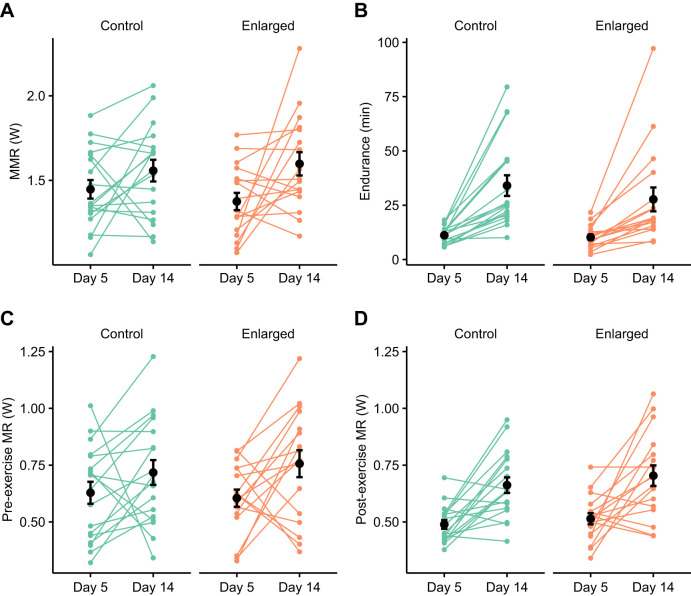
**Effect of brood size manipulation on exercise capacity of male blue tits.** (A) Maximum metabolic rate (MMR), (B) endurance, (C) pre-exercise metabolic rate (MR) and (D) post-exercise MR measured in male blue tits on the day of brood size manipulation (day 5) and 9 days after (day 14) with control and experimentally enlarged broods. Each individual bird is represented by one line. Black points and error bars show group means and s.e.m.

After 9 days of feeding nestlings, we found that MMR, endurance, pre-exercise metabolic rate (MR) and post-exercise MR increased to a similar extent in males with experimentally enlarged broods and in males with unmanipulated control brood size (Fig. 2, Table 2). MMR increased by 16% between day 5 and day 14 and endurance increased by 157%, from a mean (±s.e.m.) of 9.8±1.1 min on day 5 to 25.1±1.1 min on day 14. Pre-exercise MR increased by 18% and post-exercise MR increased by 38% between day 5 and day 14. Body mass was positively related to MMR but not to endurance or RMR. The random intercept, ID, explained a significant amount of variation in endurance (intraclass correlation coefficient, ICC=0.68) but we did not find a significant effect of ID on MMR (ICC=0), pre-exercise MR (ICC=0.15) or post-exercise MR (ICC=0.12). We did not find an effect of holding time on any parameter. Birds decreased in body mass by a mean of 0.1 g, or about 1% of body mass, from day 5 to day 14. Feeding frequency on day 13 and nestling mass on day 14 did not differ significantly between males with enlarged and control nests (*t*-test, feeding frequency: *t*_28_=−1.77, *P*=0.088, mean±s.e.m. feeding frequency control: 20.1±1.7 h^−1^, enlarged: 24.2±1.6 h^−1^, nestling mass: *t*_33_=1.23, *P*=0.23, mean±s.e.m. mass control: 11.3±0.1 g, enlarged: 11.0±0.2 g).

**Table 2.** Results of linear mixed-effects models (LMM) of male blue tit body mass, maximum metabolic rate (MMR), endurance, pre-exercise metabolic rate (MR) and post-exercise MR before (day 5 after hatching) and after (day 14 after hatching) brood size manipulation

Parameter	Estimate (variance)	s.e. (s.d.)	d.f.	*F*/LRT	*P*
**Body mass (g)**					
Brood size category			1,33.01	0.013	0.91
Control	11.36	0.11			
Enlarged	11.34	0.11			
Day			1, 32.6	7.22	**0.011**
5	11.41	0.081			
14	11.28	0.081			
Holding time	−0.23	0.12	1, 37.45	3.85	0.057
Brood size×day			1, 32.15	0.12	0.74
Nest ID (random)	(0.19)	(0.44)	1	(39.66)	**<0.001**
Residual	(0.037)	(0.19)			
**MMR (ml O_2_ min^−1^)**					
Brood size			1, 64	0.017	0.90
Control	1.50	0.038			
Enlarged	1.49	0.039			
Day			1, 64	15.63	**<0.001**
5	1.38	0.039			
14	1.60	0.039			
Holding time	−0.17	0.10	1, 64	2.75	0.10
Body mass	0.21	0.058	1, 64	13.41	**<0.001**
Brood size×day			1, 64	0.97	0.33
Nest ID (random)	(0.00)	(0.00)	1	(0.00)	1
Residual	(0.051)	(0.23)			
**Endurance (min)**					
Brood size			1, 32.17	1.76	0.19
Control	17.38	1.12			
Enlarged	14.13	1.12			
Day			1, 35.63	136.83	**<0.001**
5	9.77	1.10			
14	25.12	1.10			
Holding time	1.24	1.20	1, 44.84	1.34	0.25
Body mass	1.04	1.17	1, 55.94	0.060	0.81
Brood size×day			1, 31.87	0.60	0.44
Nest ID (random)	(0.039)	(0.20)	1	(19.89)	**<0.001**
Residual	(0.018)	(0.14)			
**Pre-exercise MR (ml O_2_ min^−1^)**					
Brood size			1, 31.52	0.017	0.90
Control	0.67	0.038			
Enlarged	0.68	0.039			
Day			1, 35.07	5.50	**0.025**
5	0.62	0.037			
14	0.73	0.037			
Holding time	−0.056	0.095	1, 62.70	0.34	0.56
Body mass	−0.080	0.058	1, 38.65	1.88	0.18
Brood size×day			1, 32.10	0.48	0.49
Nest ID (random)	(0.0067)	(0.082)	1	0.70	0.40
Residual	(0.039)	(0.20)			
**Post-exercise MR (ml O_2_ min^−1^)**					
Brood size			1, 27.34	1.00	0.33
Control	0.58	0.024			
Enlarged	0.61	0.025			
Day			1, 30.86	33.92	**<0.001**
5	0.50	0.023			
14	0.69	0.023			
Holding time	−0.072	0.061	1, 63.05	1.38	0.25
Body mass	−0.028	0.037	1, 34.01	0.57	0.45
Brood size×day			1, 27.96	0.08	0.78
Nest ID (random)	(0.0021)	(0.045)	1	(0.30)	0.58
Residual	(0.016)	(0.13)			

Estimated marginal means of factors and slopes of co-variates (estimate) or variance of random effects and residuals, standard error (s.e.) of fixed effects or standard deviation (s.d.) of random effects and residuals in parentheses, *F*-statistic (or likelihood ratio test, LRT, for random effects) and levels of significance are shown (*P*<0.05 in bold).

### Effect of brood size manipulation and age on exercise capacity

**Fig. 3.**
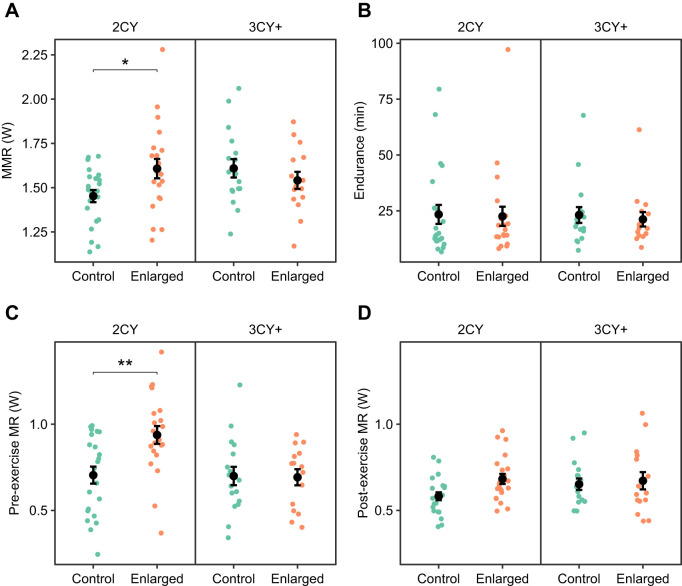
**Effect of brood size manipulation and age on exercise capacity of male blue tits.** (A) MMR, (B) endurance and (C,D) resting metabolic rate before (pre-exercise MR; C) and after (post-exercise MR; D) an exercise test in male blue tits. Birds were grouped by age as 2nd calendar year (2CY) or 3rd or greater calendar year (3CY+). Birds were measured on day 14 after chicks hatched, 9 days after brood size manipulation (control or enlarged). Black points and error bars represent means and s.e.m. Asterisks indicate significance of pairwise comparisons of emmeans using the Tukey method (*P<0.05, **P<0.01).

We found that the effect of the brood size manipulation on MMR and pre-exercise MR differed depending on age (brood size×age interaction; Fig. 3, Table 3). In 2CY birds, MMR was 10% higher in parents of enlarged broods compared with control broods. Similarly, pre-exercise MR was 33% higher in enlarged nests than in control nests of 2CY birds. Older calendar year birds showed no difference in MMR or pre-exercise MR with brood size manipulation. Post-exercise MR showed a similar trend to MMR and pre-exercise MR, but we did not find a significant interaction between brood size category and age. Birds with enlarged nests had 10% higher post-exercise MR than birds with control nests overall. MMR, but not pre-exercise MR, post-exercise MR or endurance, was positively related to body mass. We did not find an effect of brood size category on endurance, but endurance was significantly higher in 2024 than in 2023, by 85%. We did not find a significant effect of holding time on MMR, endurance, pre-exercise MR or post-exercise MR.

**Table 3.** Linear models (LM) of the effect of brood size manipulation on male blue tit MMR, endurance, pre-exercise MR and post-exercise MR

Parameter	Estimate	s.e.	d.f.	*F*	*P*
**MMR (ml O_2_ min^−1^)**					
Brood size category		0.059	1, 68	6.78	**0.011**
Age		0.063	1, 68	3.54	*0.064*
Holding time	−0.051	0.069	1, 68	0.56	0.46
Body mass	0.15	0.044	1, 68	11.31	**0.001**
Year			1, 68	1.74	0.19
2023	1.52	0.033			
2024	1.59	0.034			
Brood size×age			1, 68	4.85	**0.031**
2CY					
Control	1.47	0.041			C−E: **0.011**
Enlarged	1.62	0.043			
3CY+					
Control	1.59	0.047			C−E: 0.522
Enlarged	1.54	0.050			
**Endurance (log_10_min)**					
Brood size			1, 68	0.050	0.82
Control	19.23	1.09			
Enlarged	18.66	1.09			
Age			1, 68	0.046	0.83
2CY	19.95	1.09			
3CY+	17.99	1.10			
Holding time	0.98	1.20	1, 68	0.0066	0.94
Body mass	1.05	1.13	1, 68	0.15	0.70
Year			1, 68	21.2	**<0.001**
2023	13.90	1.09			
2024	25.76	1.09			
Brood size×age			1, 68	0.29	0.59
**Pre-exercise MR (ml O_2_ min^−1^)**					
Brood size			1, 68	11.06	**0.0014**
Age			1, 68	0.049	0.82
Holding time	−0.0053	0.079	1, 68	0.0045	0.95
Body mass	−0.092	0.050	1, 68	3.35	*0.072*
Year			1, 68	0.45	0.50
2023	0.78	0.038			
2024	0.74	0.039			
Brood size×age			1, 69	5.83	**0.018**
2CY					
Control	0.70	0.048			C−E: **0.001**
Enlarged	0.93	0.050			
3CY+
Control	0.72	0.054			C−E: 0.77
Enlarged	0.70	0.058			
**Post-exercise MR (ml O_2_ min^−1^)**					
Brood size			1, 68	5.24	**0.025**
Control	0.62	0.023			
Enlarged	0.68	0.024			
Age			1, 68	1.46	0.23
2CY	0.64	0.066			
3CY+	0.65	0.074			
Holding time	−0.021	0.050	1, 68	0.17	0.68
Body mass	−0.0064	0.032	1, 68	0.041	0.84
Year			1, 68	3.18	*0.079*
2023	0.62	0.024			
2024	0.68	0.025			
Brood size×age			1, 68	1.72	0.19

Estimated marginal means of factors and slopes of co-variates (estimate), standard error (s.e.), *F*-statistic and levels of significance are shown (*P*<0.05 in bold and *P*<0.1 in italics). Significance levels of pairwise comparisons in *emmeans* are shown for significant interaction terms. C−E: control−enlarged.

**Fig. 4.**
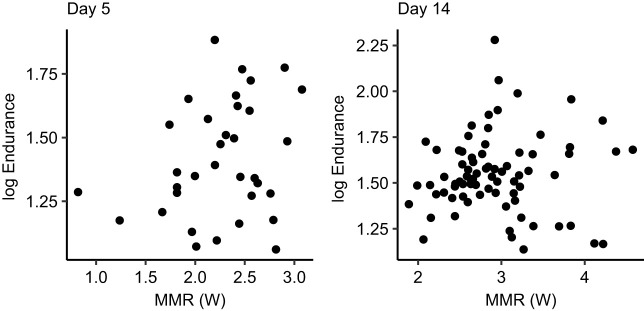
**MMR did not influence endurance in breeding blue tits, measured when nestlings were 5 or 14 days old.** Values are log endurance (measured in min).

MMR was not related to endurance in birds on day 5 (linear regression, *F*_1,33_=2.22, *P*=0.15) or day 14 (*F*_1,74_=0.18, *P*=0.67; Fig. 4), meaning that birds with longer endurance times kept up a similar level of exertion to those that became tired earlier.

### Effect of brood size manipulation on nestling biometrics

**Fig. 5.**
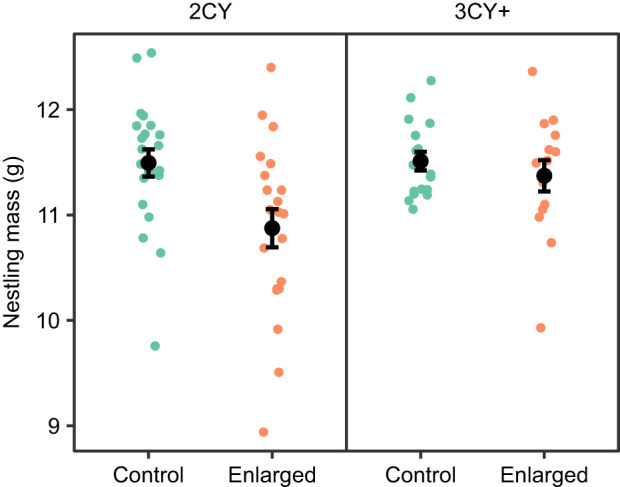
**Effect of brood size manipulation on nestling biometrics.** Nests are grouped by male parent age, 2nd calendar year (2CY) or 3rd or greater calendar year (3CY+), and brood size: control or enlarged. Points represent the mean nestling mass of one nest. Black points and error bars show group means and s.e.m.

Nestlings in control nests had 2.8% higher mass than nestlings in enlarged nests (Fig. 5, Table 4). We did not find a difference between enlarged and control nestling mass depending on the male parent's age (brood size×age interaction, *P*=0.1). Nestlings were 0.45±0.15 g (mean±s.e.m.) heavier in 2023 than in 2024. Nestling wing length tended to be shorter in enlarged broods (*P*=0.08), but the difference was small (mean±s.e.m. difference: 0.73±0.39 mm) (Table S1). We did not find an effect of brood size, parent age, hatch date or year on nestling tarsus length (Table S1).

**Table 4.** Results of linear models (LM) of the effects of a brood size manipulation on nestling blue tit mass measured 9 days later, when they were 14 days old

Parameter	Estimate	s.e.	d.f.	*F*	*P*
**Nestling body mass (g)**					
Brood size			1, 68	8.41	**0.0050**
Control	11.46	0.099			
Enlarged	11.15	0.10			
Parent age			1, 68	0.059	0.81
2CY	11.16	0.095			
3CY+	11.44	0.11			
Day 5 nestling mass	0.24	0.16	1, 68	2.36	0.13
Hatch date (April day)	−0.029	0.018	1, 68	2.52	0.12
Year			1, 68	9.22	**0.0034**
2023	11.53	0.10			
2024	11.08	0.10			
Brood size×parent age			1, 68	2.85	*0.096*

Estimated marginal means of factors and slopes of co-variates (estimate), standard error (s.e.), *F*-statistic and levels of significance are shown (*P*<0.05 in bold and *P*<0.1 in italics).

## DISCUSSION

### Phenotypic flexibility of exercise capacity

The results of this study show that exercise capacity is a flexible trait in free-living birds during the breeding season (for evidence in captive birds, see Brackenbury and El-Sayed, 1985; Butler and Turner, 1988; Zhang et al., 2015b). In line with our hypothesis, exercise capacity increased with both nestling age (Fig. 2) and experimental brood size (Fig. 3), although the latter was age specific. We propose that this was due to a training effect, as parental effort is expected to increase with both nestling age (Barba et al., 2009; Blondel et al., 1991; Grundel, 1987) and brood size (García-Navas and Sanz, 2010; Nur, 1984). Thus, our results support the hypothesis that exercise capacity is flexible and increases with parental workload in young birds, but the response to a brood size manipulation in older birds may be more nuanced.

Exercise training and exercise capacity in birds have been linked to physiological adjustments involving oxygen transport (Bury et al., 2019; Yap et al., 2021), lipid catabolism (Price et al., 2022; Zhang et al., 2015a), skeletal muscle enlargement (Petit and Vézina, 2014; Zhang et al., 2015b, 2018), mitochondrial content (Butler, 1991) and anti-oxidative capacity (Larcombe et al., 2010). These changes are thought to enhance exercise performance and to allow for a greater sustained workload over longer periods of time. While our study did not test whether an increase in exercise capacity influenced reproductive success, it is plausible that phenotypic flexibility in exercise capacity is an adaptive response to temporal variation in workload, minimizing potential costs of maintaining an unnecessarily high exercise capacity if workload decreases.

Consistent with the increase in MMR, pre- and post-exercise MR also increased with nestling age (Fig. 2). Post-exercise MR increased with experimental brood size across ages, while MMR and pre-exercise MR only increased in 2CY birds (Fig. 3). Feeding more chicks could require a higher daily energy expenditure, which would in turn necessitate a higher rate of self-feeding (Nilsson, 2002). The central organs make up a large component of the energy needed to function at rest (Suarez and Darveau, 2005). Digestive organ size is phenotypically flexible in birds and is positively related to both energy intake rate and RMR (Lindström et al., 1999). Therefore, birds with enlarged broods may have had increased energy expenditure at rest because more energy was needed to support larger organs in the alimentary tract that are involved in digestion and excretion of waste products (Barceló et al., 2017; Daan et al., 1990).

MMR, pre- and post-exercise MR were not repeatable within individuals, which suggests that while the mean estimates of MR increased between days 5 and 14, individuals showed variation in their metabolic response to increased work rate. This might be due to physiological constraints hindering a further increase, nutritional status or local variation in environmental factors. Interestingly, endurance was highly repeatable, suggesting that endurance might be a heritable trait determining an individual's investment in work, e.g. current reproduction.

Endurance was nearly twice as high on day 14 in 2024, when birds were tested in the wheel for the second time, compared with that on day 14 in 2023, when birds were naive to the wheel. However, we do not believe that learning or acclimation to the hop-flutter wheel was the main explanation for our results. Naive day 14 birds in 2023 had a significantly higher endurance than naive day 5 birds in 2024 (*t*-test: *t*_74_=3.68, *P*<0.001; mean±s.e.m.: 2023 day 14: 15.6±1.0 min, 2024 day 5: 10.7±0.8 min), which points to a true effect of endurance training, rather than an effect of habituation. As endurance was highly repeatable within individuals, differences in endurance between 2023 and 2024 could be explained by selective disappearance between the two years, as 2024 had roughly half the number of 2CY birds (*N*=94) as in 2023 (*N*=212), while the number of 3CY+ birds in the population was relatively stable (2023: *N*=112; 2024: *N*=99). Alternatively, a higher mean nestling mass in the population in 2023 (*t*-test, *t*_198_=2.28, *P*=0.024; mean±s.e.m., 2023: 11.544±0.053 g, 2024: 11.321±0.091 g; Fig. S1) suggests a lower resource availability in 2024, which could result in an even stronger training effect for endurance during this year.

### Age-dependent effects of brood size manipulation

Our results suggest that 2CY parents of control broods invested sub-maximally in parental care, as those caring for enlarged broods had higher exercise capacity (Fig. 3). This could be an evolutionary strategy to optimize lifetime fitness (Drent and Daan, 1980; Williams, 2012). Reproductive success is known to peak at age 2–4 years in blue tits, after the first breeding season but before senescence sets in (Auld and Charmantier, 2011; Bonamour et al., 2020), and the probability of extra-pair paternity increases 5-fold for males between the first and subsequent breeding seasons (Schlicht and Kempenaers, 2023). An increase in parental effort during the current breeding attempt could compromise survival or future breeding success by advancing senescence (Auld and Charmantier, 2011; Boonekamp et al., 2020), impairing parasite resistance (Stjernman et al., 2004) and reducing the quality of newly grown feathers (Nilsson and Svensson, 1996). Therefore, it could be an evolutionary strategy for first-year breeders to restrict parental effort in their first breeding attempt if it increases their chances for survival and having a second breeding season.

3CY+ parents of enlarged broods had a similar exercise capacity to those with control broods (Fig. 3), but they were able to raise nestlings with a similar mass to controls, despite having five extra nestlings to feed (Fig. 5). In this study, the total brood mass on day 14 was 43% higher in enlarged broods (mean±s.e.m.: 165.7±3.6 g) than in control broods (116.0±3.01 g); thus, more food must have been brought to the enlarged broods. One possible explanation is that older birds work more efficiently as a result of their experience, or higher territory quality, compared with 2CY birds, and were able to compensate for a larger brood size without working significantly harder to feed them.

In line with this, we did not find a significant difference in feeding frequency between control and enlarged nests. However, because of variation in foraging trip distance, duration and load size, feeding frequency has been found to be poorly correlated with both parental effort and the actual amount of food brought to the nest (Williams, 2012). This makes it difficult to verify the actual parental effort in this study, as parents could increase their workload and the amount of food delivered to the brood with a minimal increase in feeding frequency if they increase the foraging effort per trip, for example by increasing load size.

Another factor that potentially could influence both male parental effort and nestling mass is female effort. While we did not measure female feeding frequency or exercise capacity, female quality is known to influence male effort (Mahr et al., 2012) and vice versa, and these behavioral effects may also be age dependent (Johnsen et al., 2005). Female compensation could contribute to why nestlings of control and enlarged nests were of similar size, even though control 2CY males had a lower exercise capacity than males with enlarged nests, and 3CY+ males in enlarged nests did not increase exercise capacity relative to control males.

### Conclusions

Our study shows that the capacity for exercise can change during the breeding season and seems to be positively related to nestling food demands. However, the response to an increased parental workload seems to be age specific, and some birds may work at a sub-maximal level. If exercise capacity gives an accurate approximation of field metabolic rate (FMR), the actual amount of work performed by parents may reflect an evolutionary strategy that balances the potential gains and losses of current and future reproductive success depending on current age, experience and resource availability.

Parental effort has often been approximated by feeding frequency, but the effects of feeding behavior such as foraging flight distance, effort, efficiency and load size are unknown when measuring feeding frequency alone. However, by measuring exercise capacity, we come closer to an understanding of how wild birds respond metabolically to an increased workload. To determine the breadth of flexibility of exercise capacity, and how well it correlates to workload during the breeding season, we suggest that future studies measure daytime FMR using methods such as accelerometers or the doubly labeled water method to verify manipulation of parental effort.

## Supplementary Material

10.1242/jexbio.251043_sup1 Supplementary information
