# Supplementary material for: Flexibility of exercise capacity during nestling feeding in blue tits
Source: J Exp Biol. 2026 Mar 31;229(7):jeb251043. doi: 10.1242/jeb.251043 (PMC13086491; doi:10.1242/jeb.251043)
Supplement: Supplementary information [file jexbio-229-251043-s1.pdf]

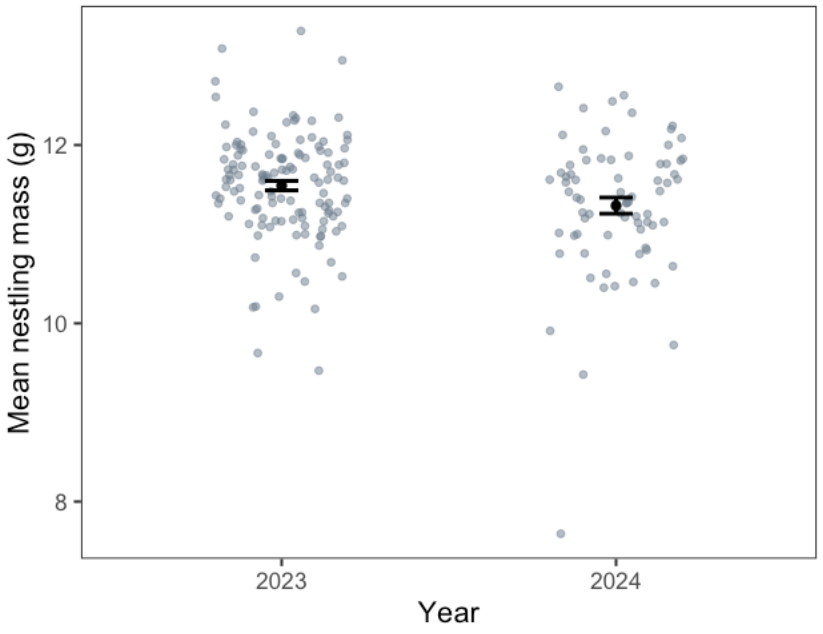

Fig. S1. Comparison of mean nestling mass in the blue tit study population in the years 2023 and 2024 and with a brood size of between 7 and 14 nestlings. Black points and error bars represent mean and SE. Mean nestling mass was significantly lower in 2024 than in 2023 (t-test, $t_{198} = 2.28$, $p = 0.024$; mean ± SE, 2023: 11.544 ± 0.053, 2024: 11.321 ± 0.091).

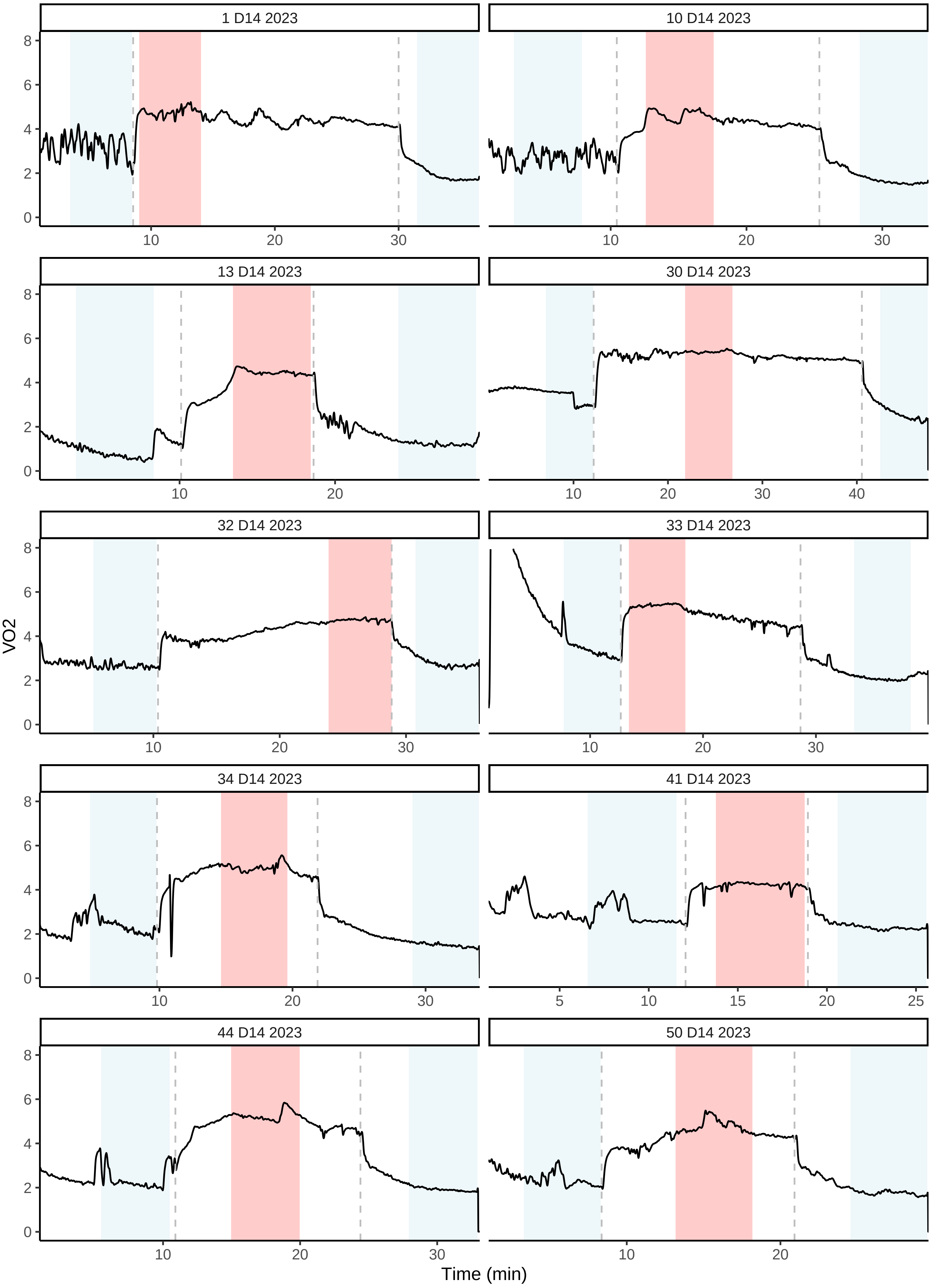
1 D14 2023
10 D14 2023
13 D14 2023
30 D14 2023
32 D14 2023
33 D14 2023
34 D14 2023
41 D14 2023
44 D14 2023
50 D14 2023
VO2
Time (min)

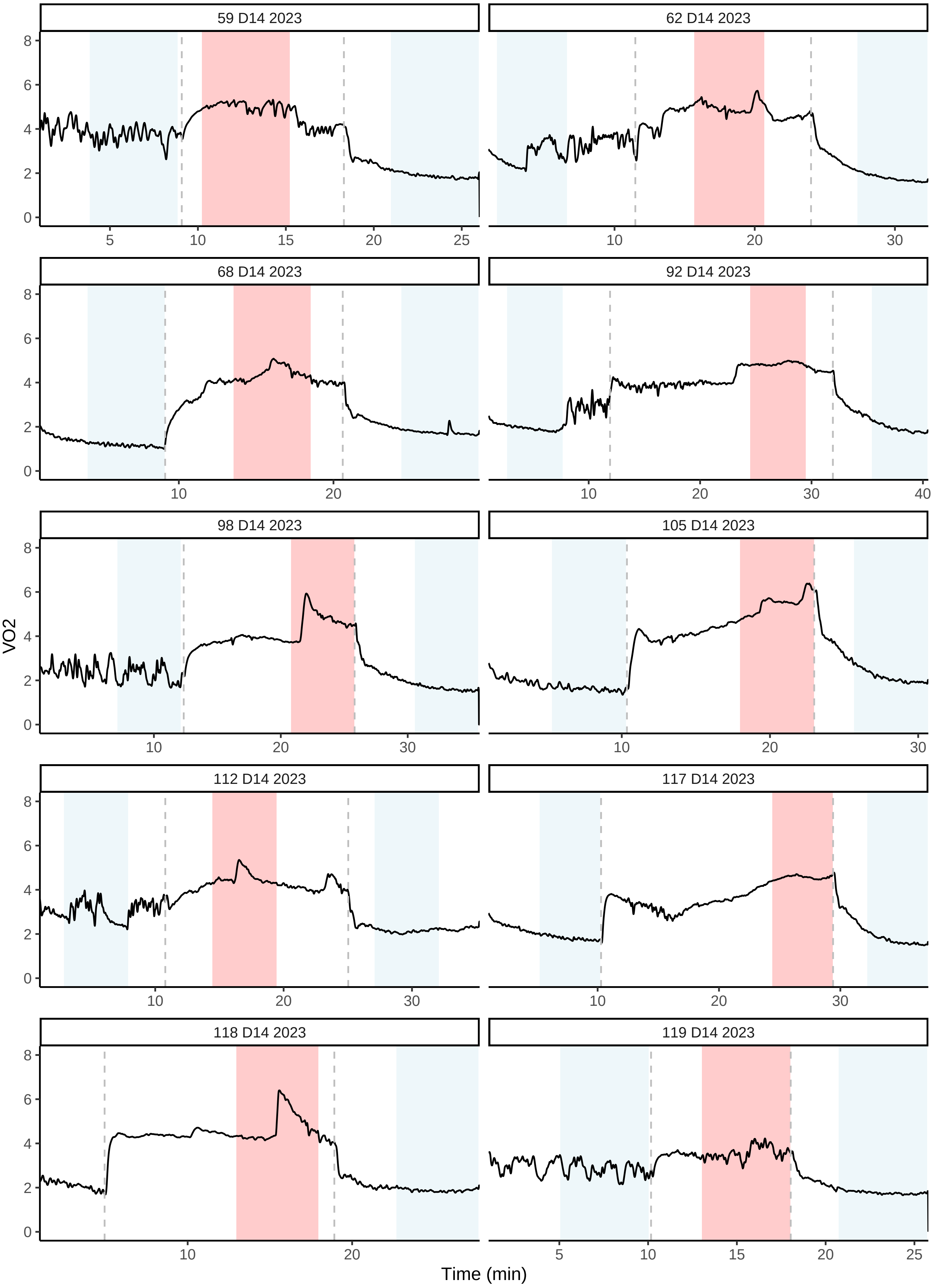
59 D14 2023
62 D14 2023
68 D14 2023
92 D14 2023
98 D14 2023
105 D14 2023
112 D14 2023
117 D14 2023
118 D14 2023
119 D14 2023
VO2
Time (min)
0
2
4
6
8
5
10
15
20
25
30
40

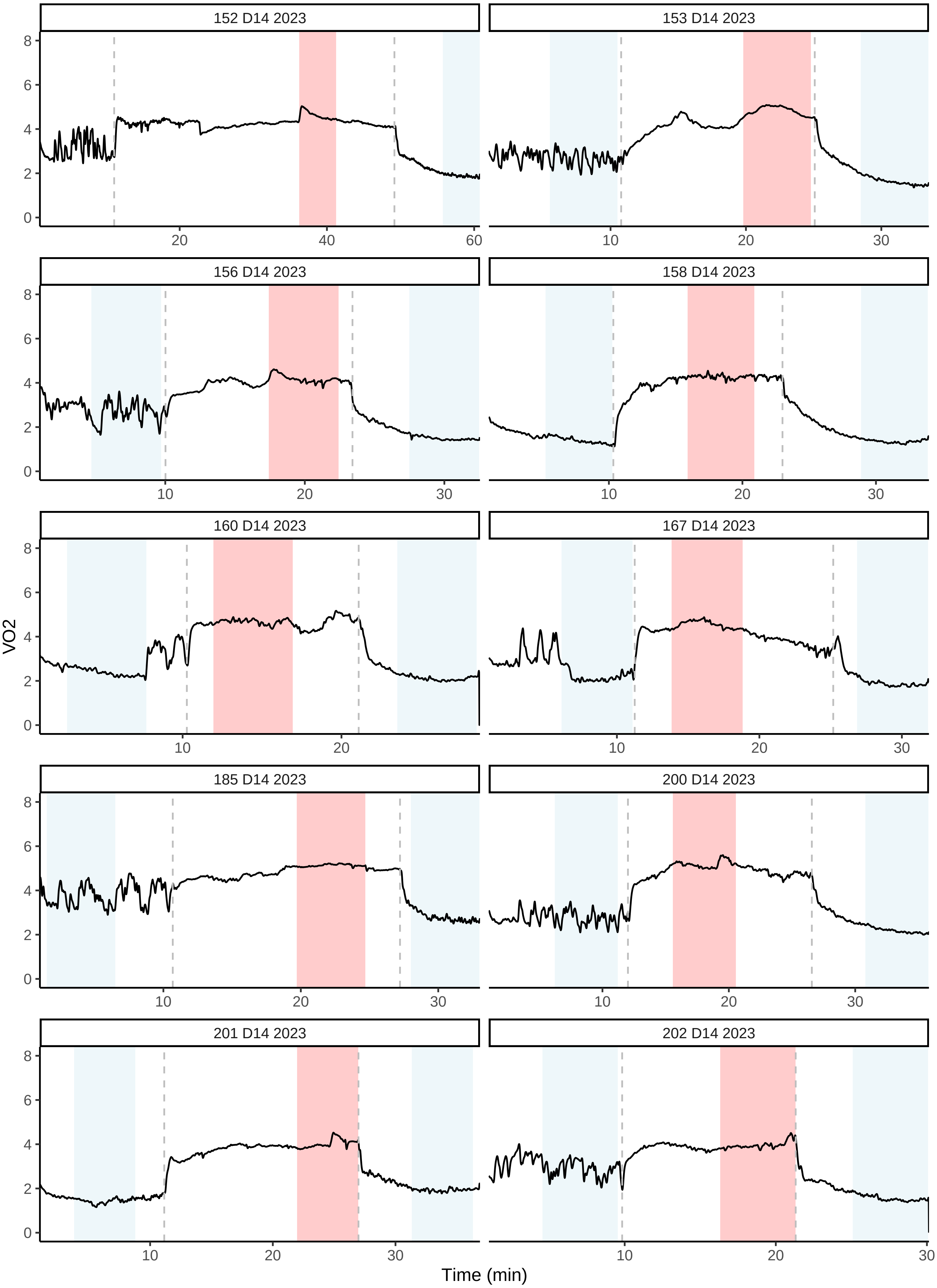

Journal of Experimental Biology • Supplementary information

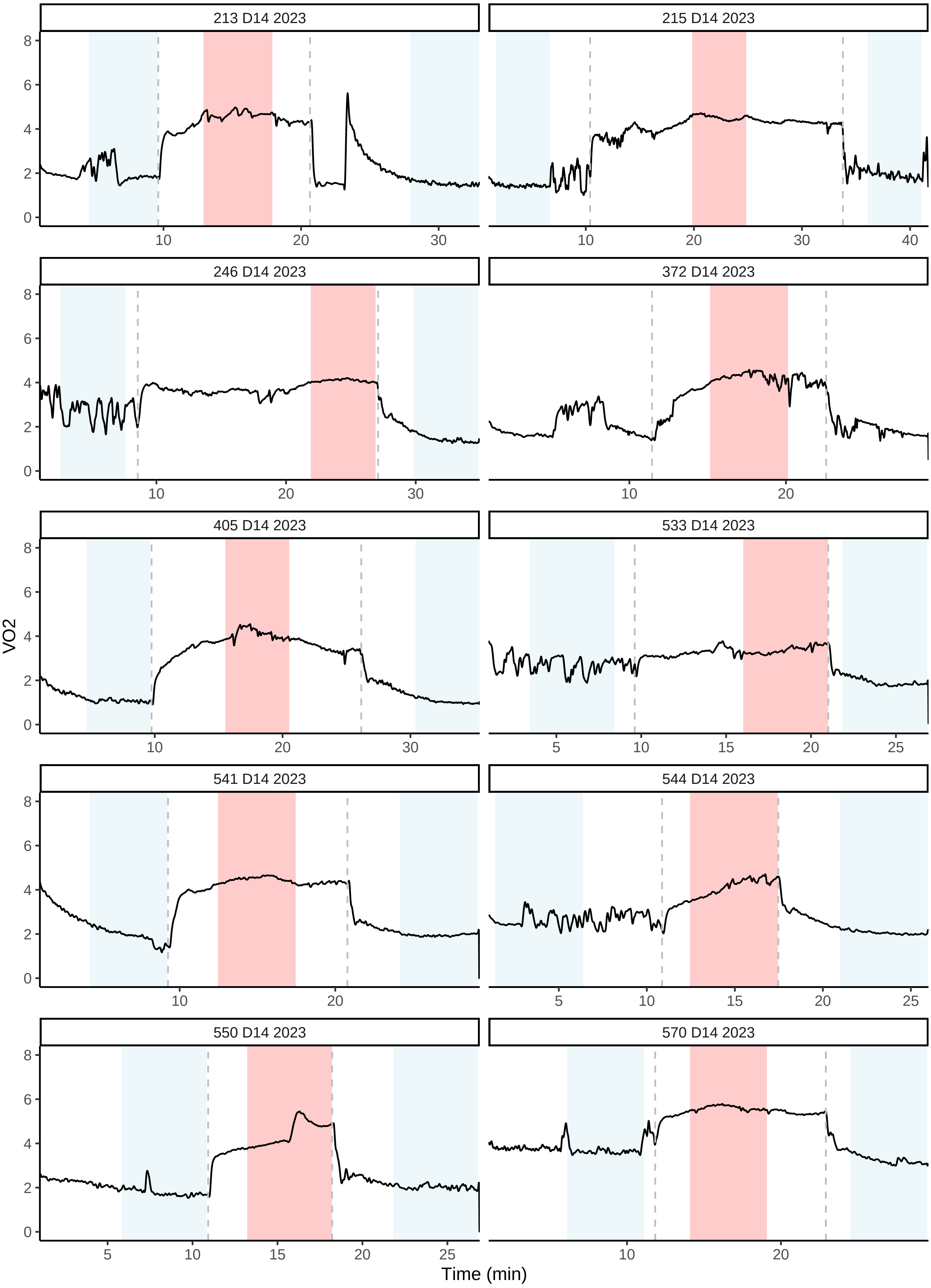
213 D14 2023
215 D14 2023
246 D14 2023
372 D14 2023
405 D14 2023
533 D14 2023
541 D14 2023
544 D14 2023
550 D14 2023
570 D14 2023
VO2
Time (min)

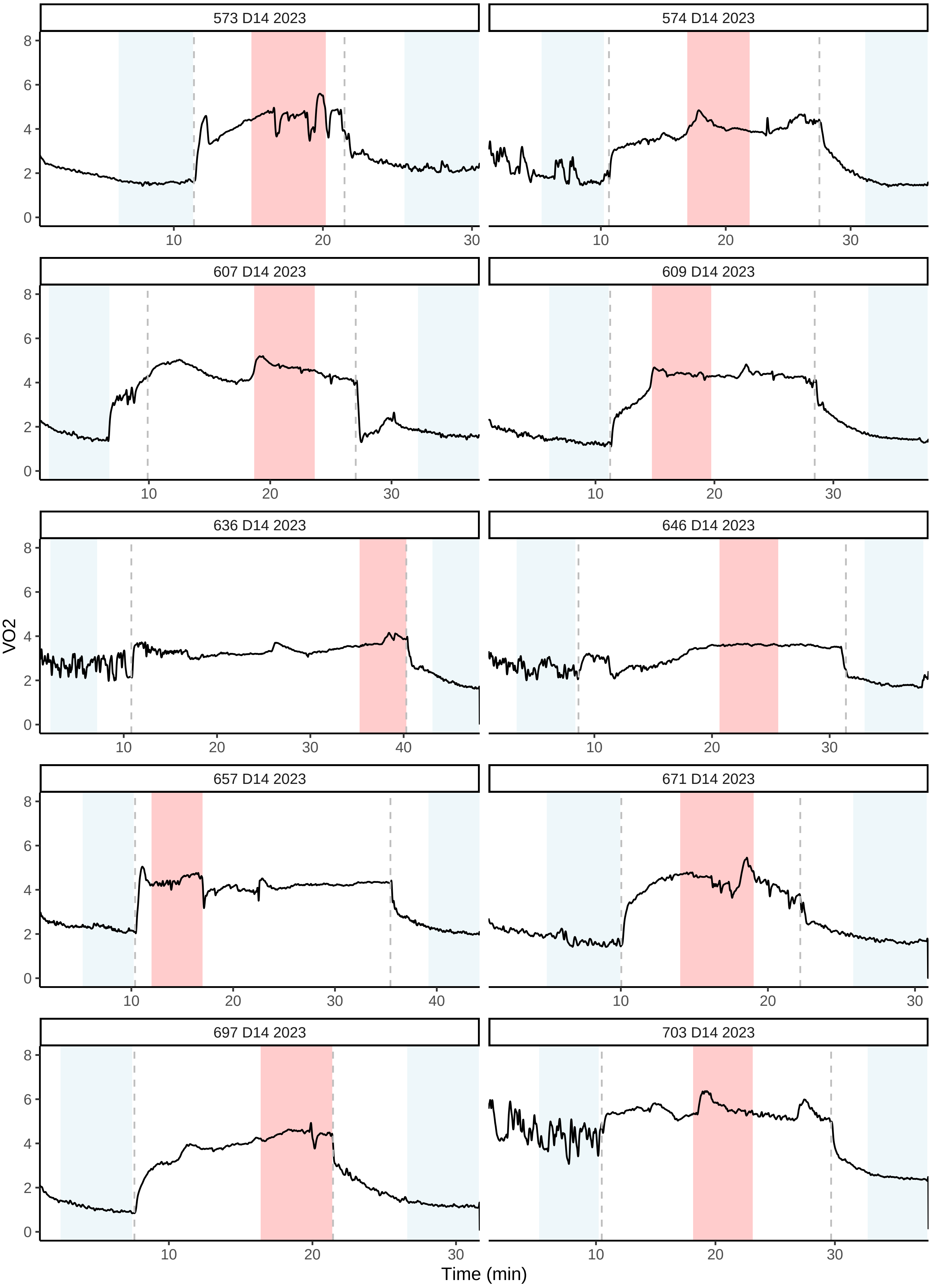
573 D14 2023
574 D14 2023
607 D14 2023
609 D14 2023
636 D14 2023
646 D14 2023
657 D14 2023
671 D14 2023
697 D14 2023
703 D14 2023
VO2
Time (min)

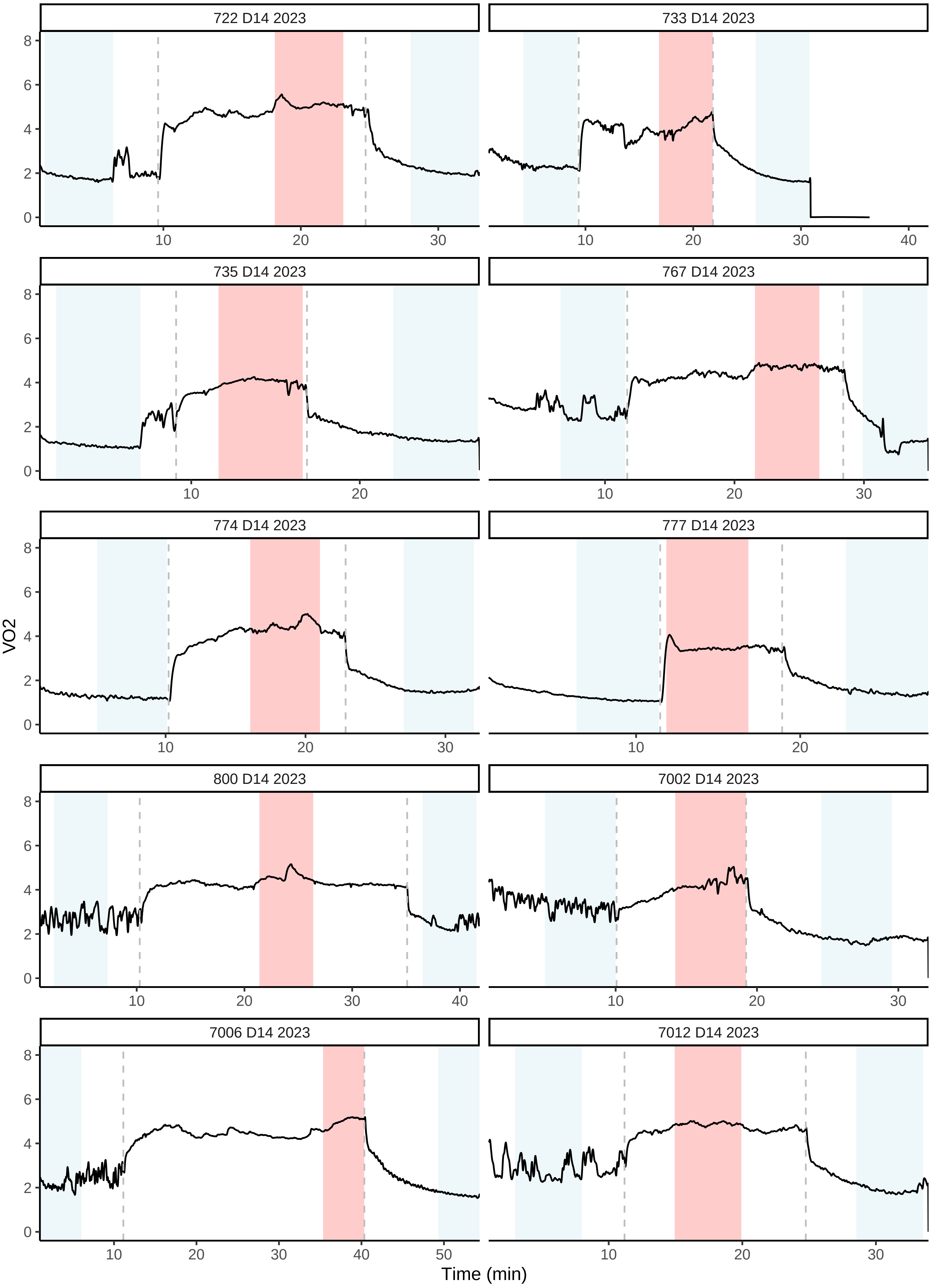

Journal of Experimental Biology • Supplementary information

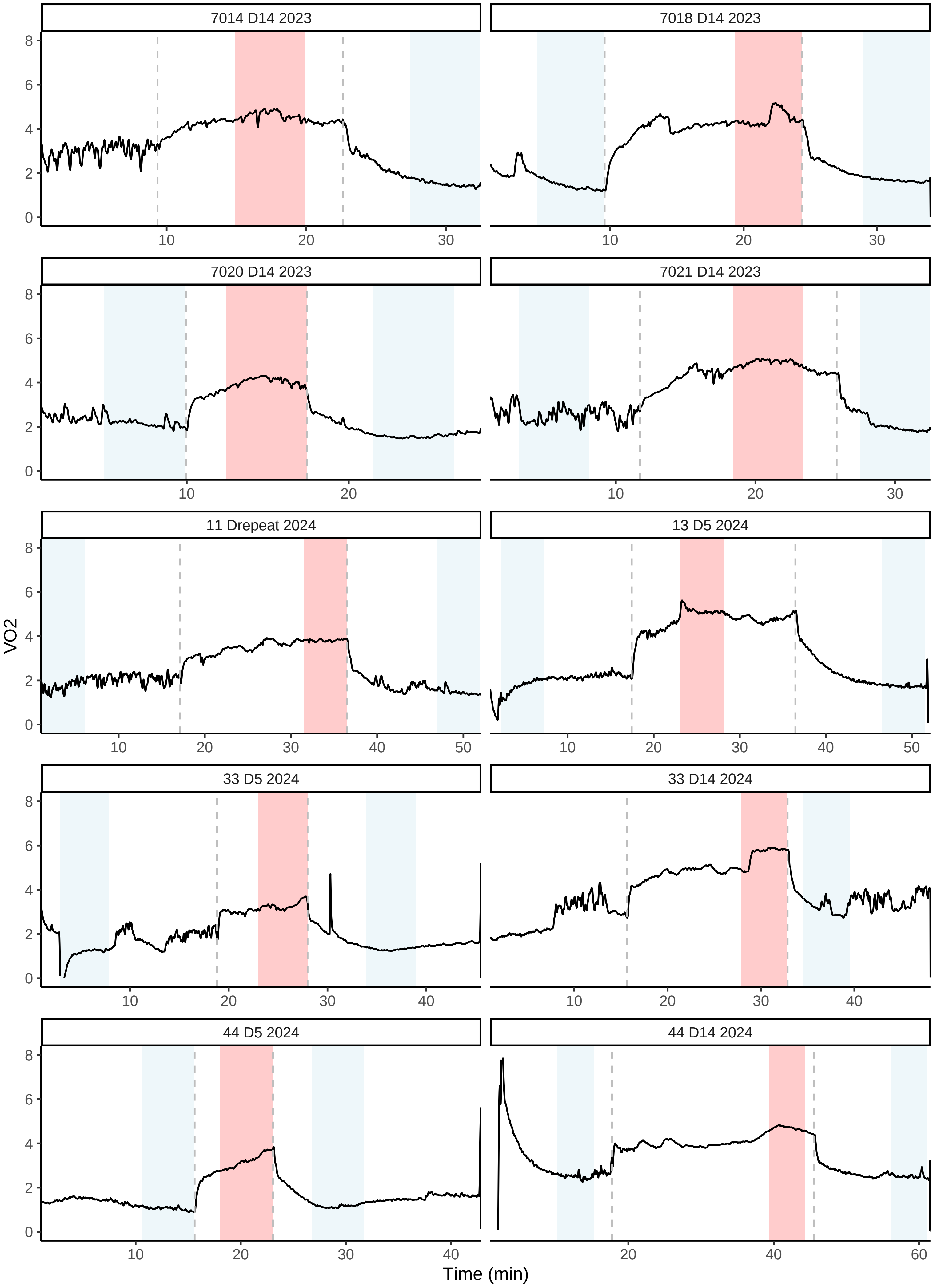

Journal of Experimental Biology • Supplementary information

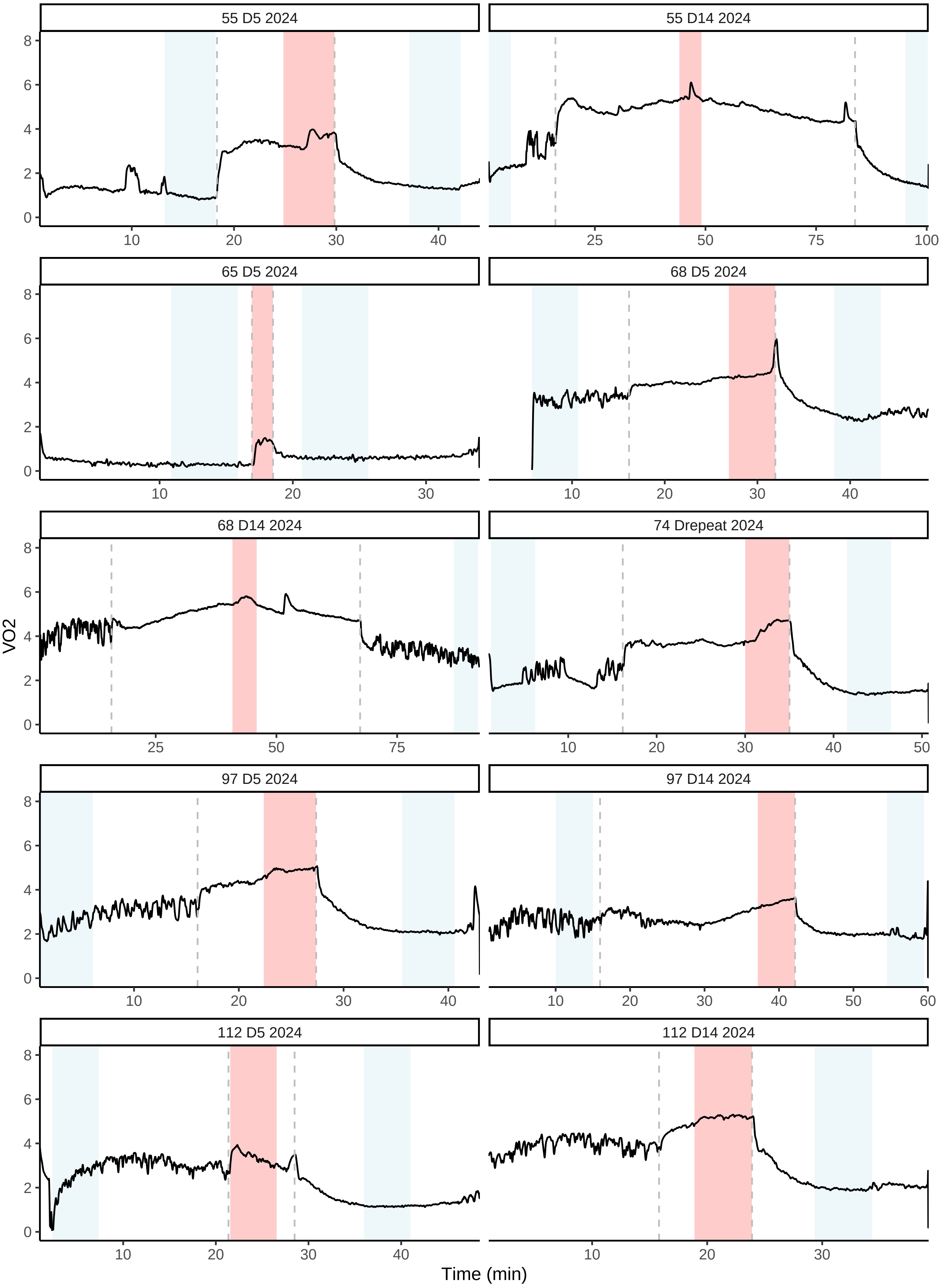

Journal of Experimental Biology • Supplementary information

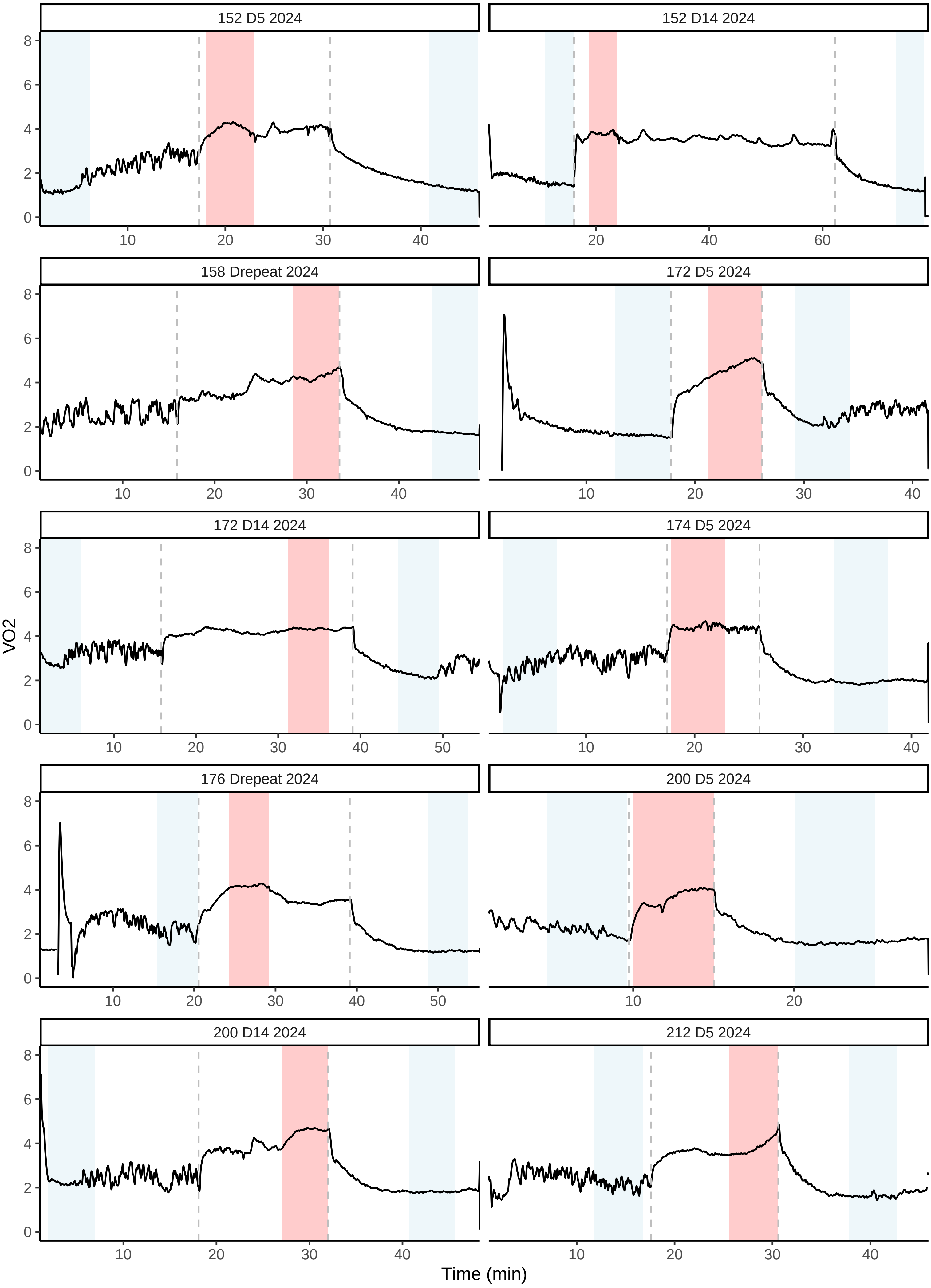

Journal of Experimental Biology • Supplementary information

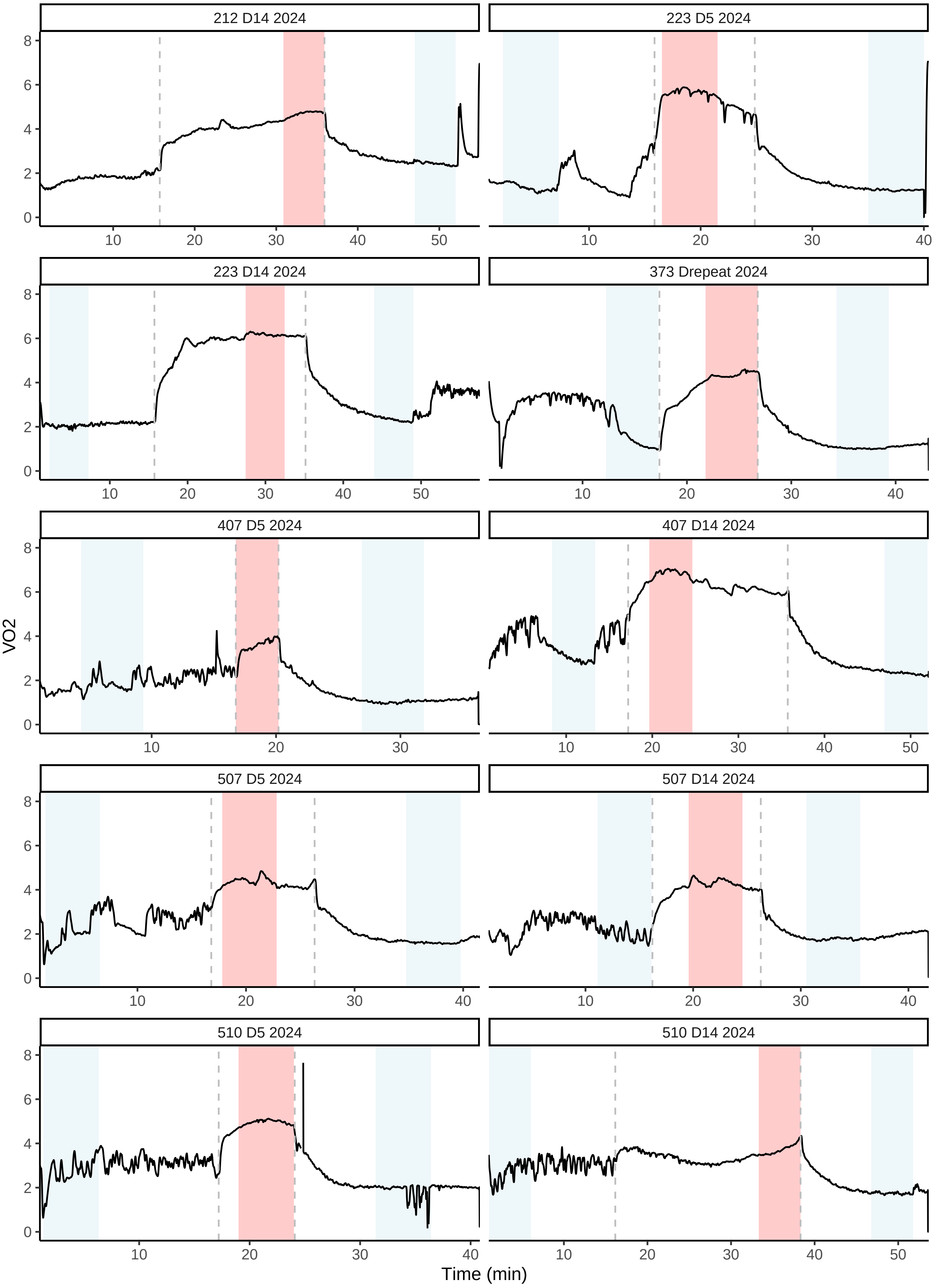

Journal of Experimental Biology • Supplementary information

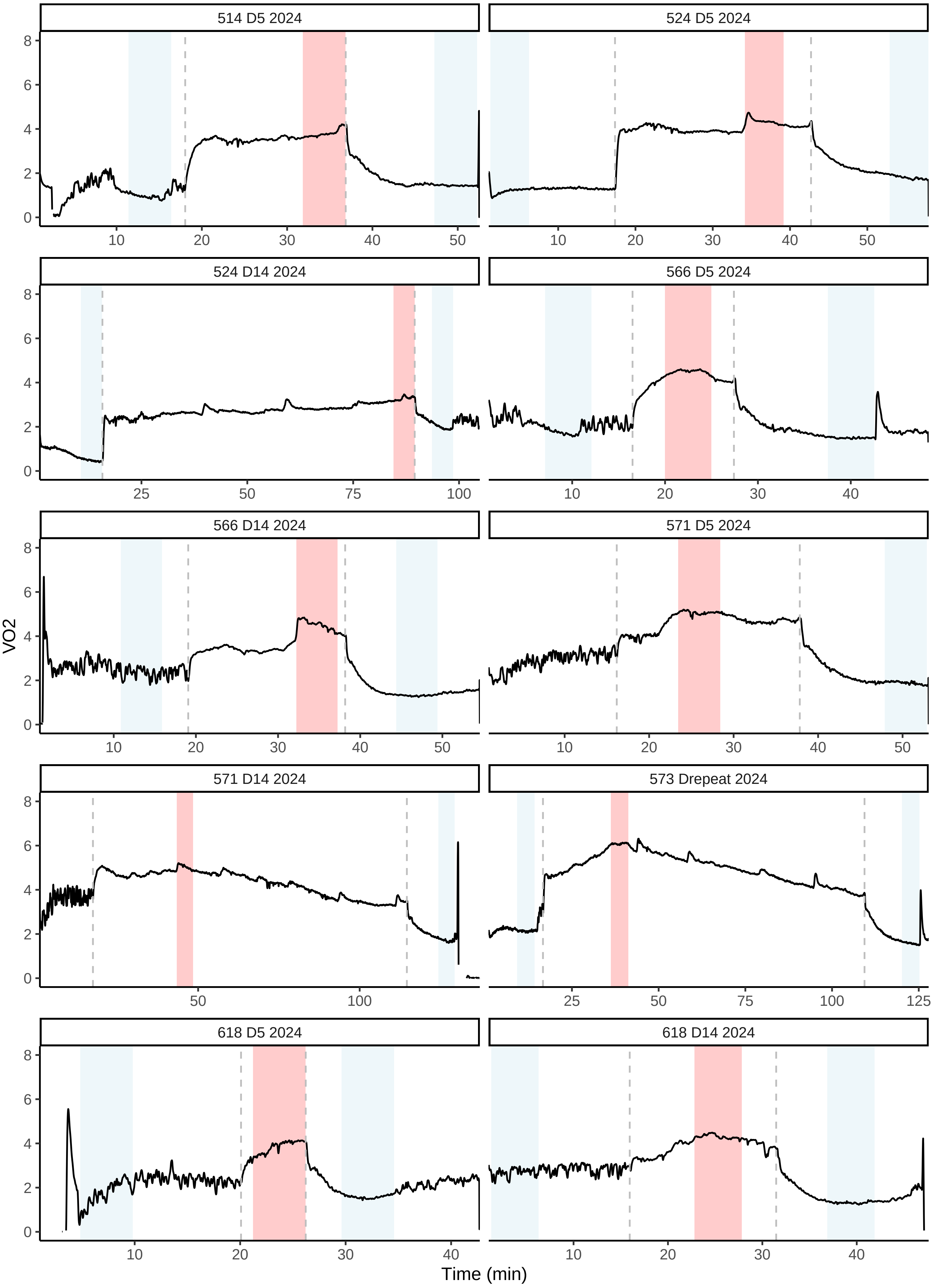

Journal of Experimental Biology • Supplementary information

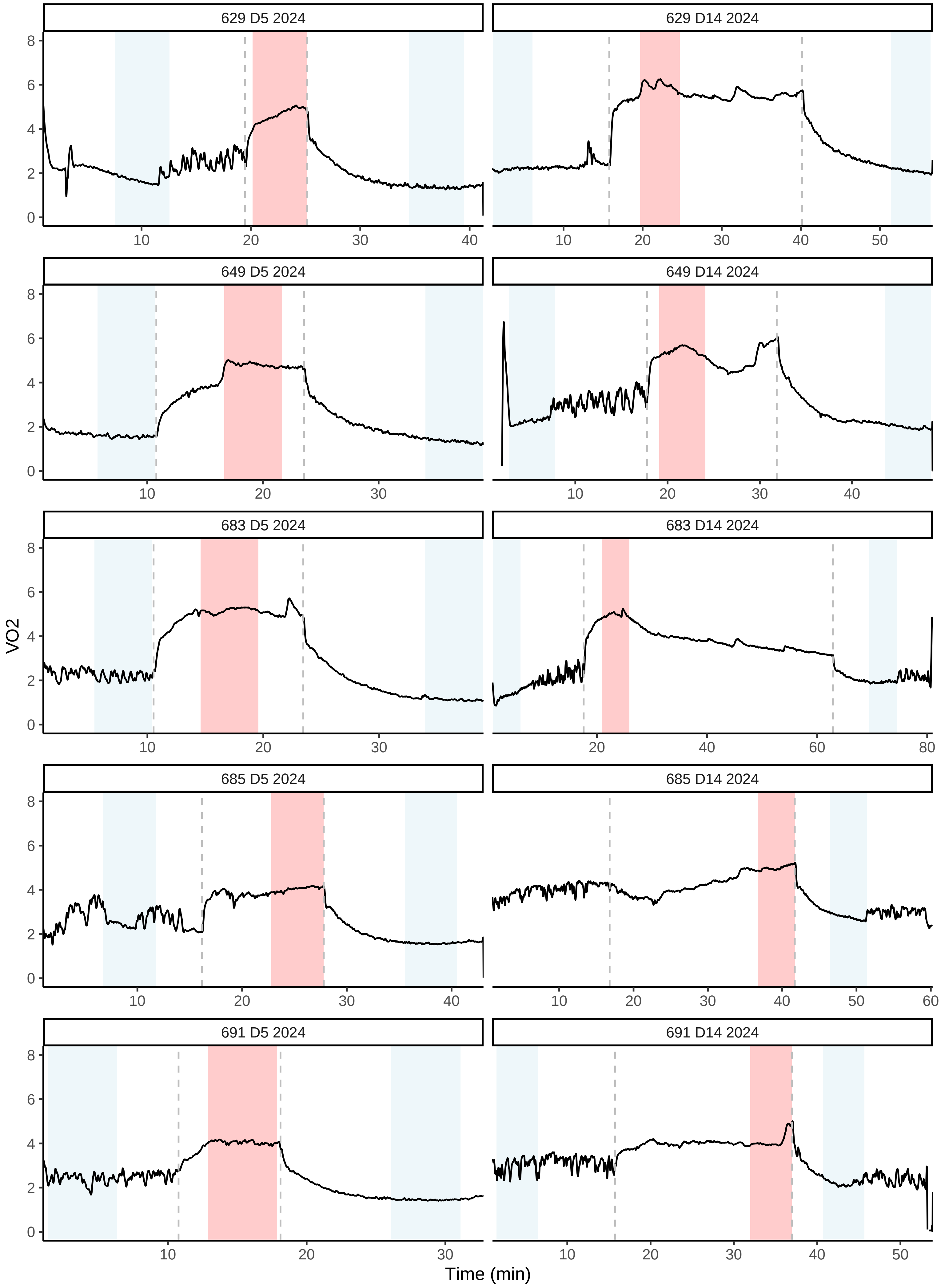

Journal of Experimental Biology • Supplementary information

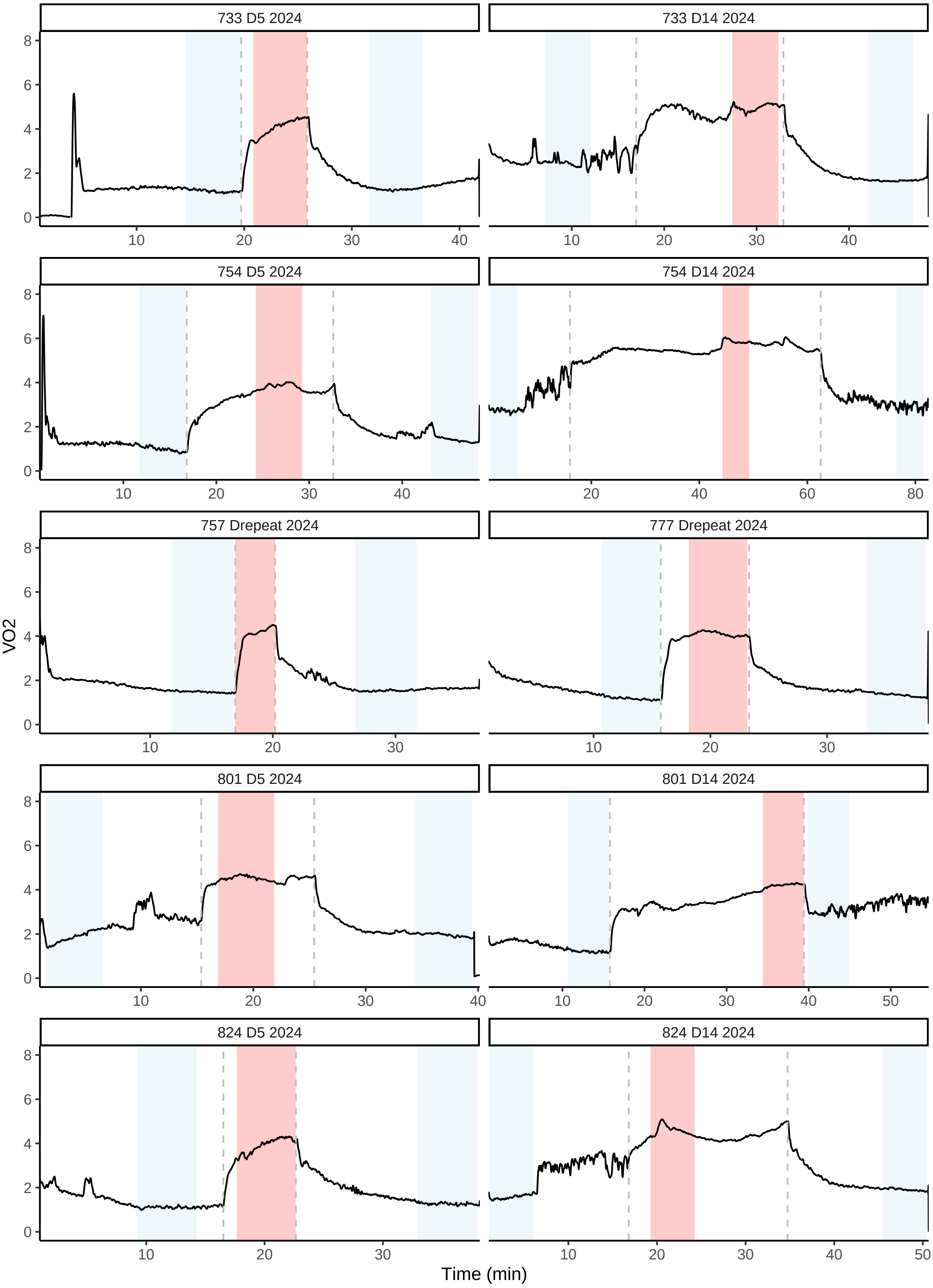

Journal of Experimental Biology • Supplementary information

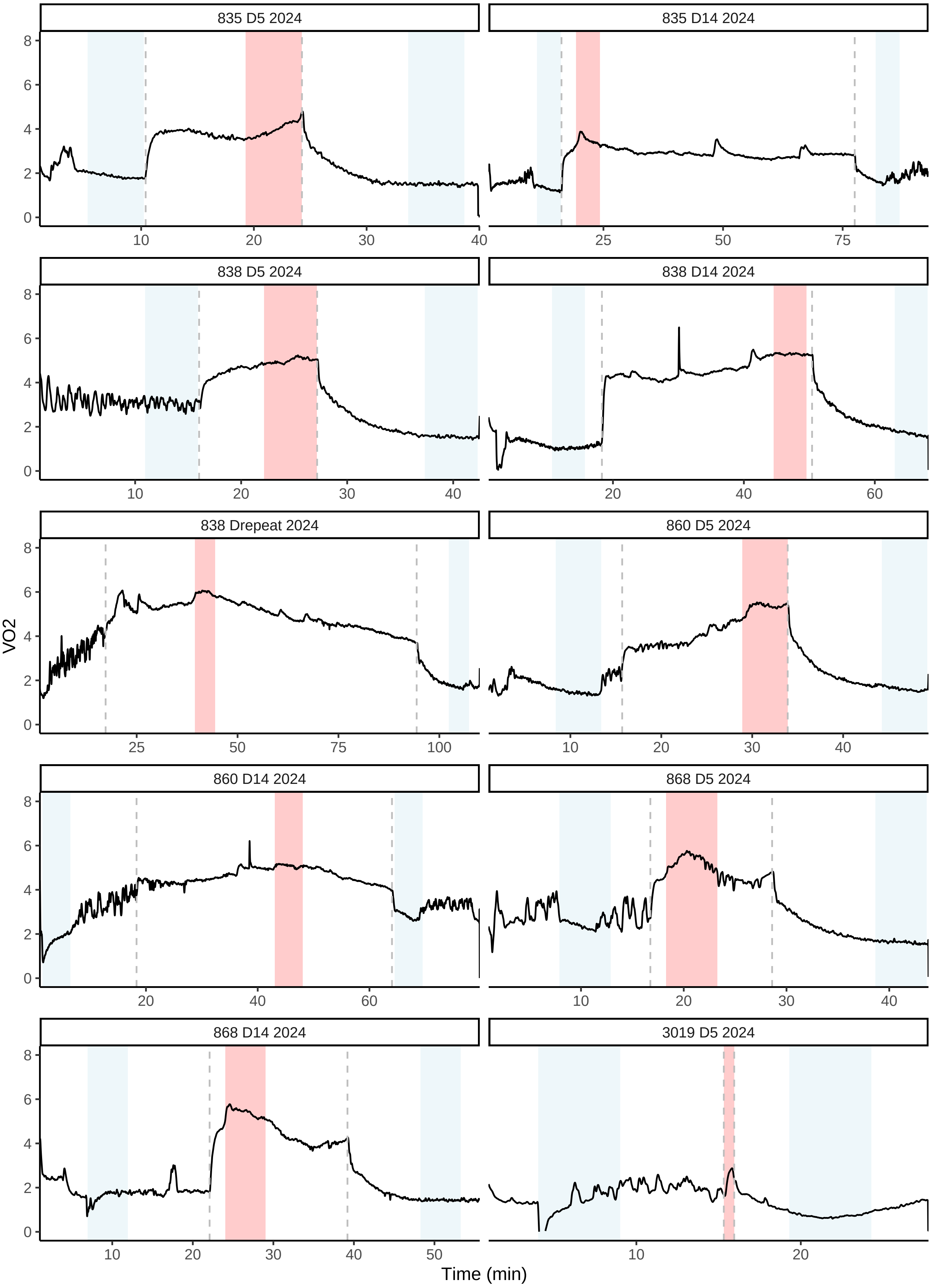

Journal of Experimental Biology • Supplementary information

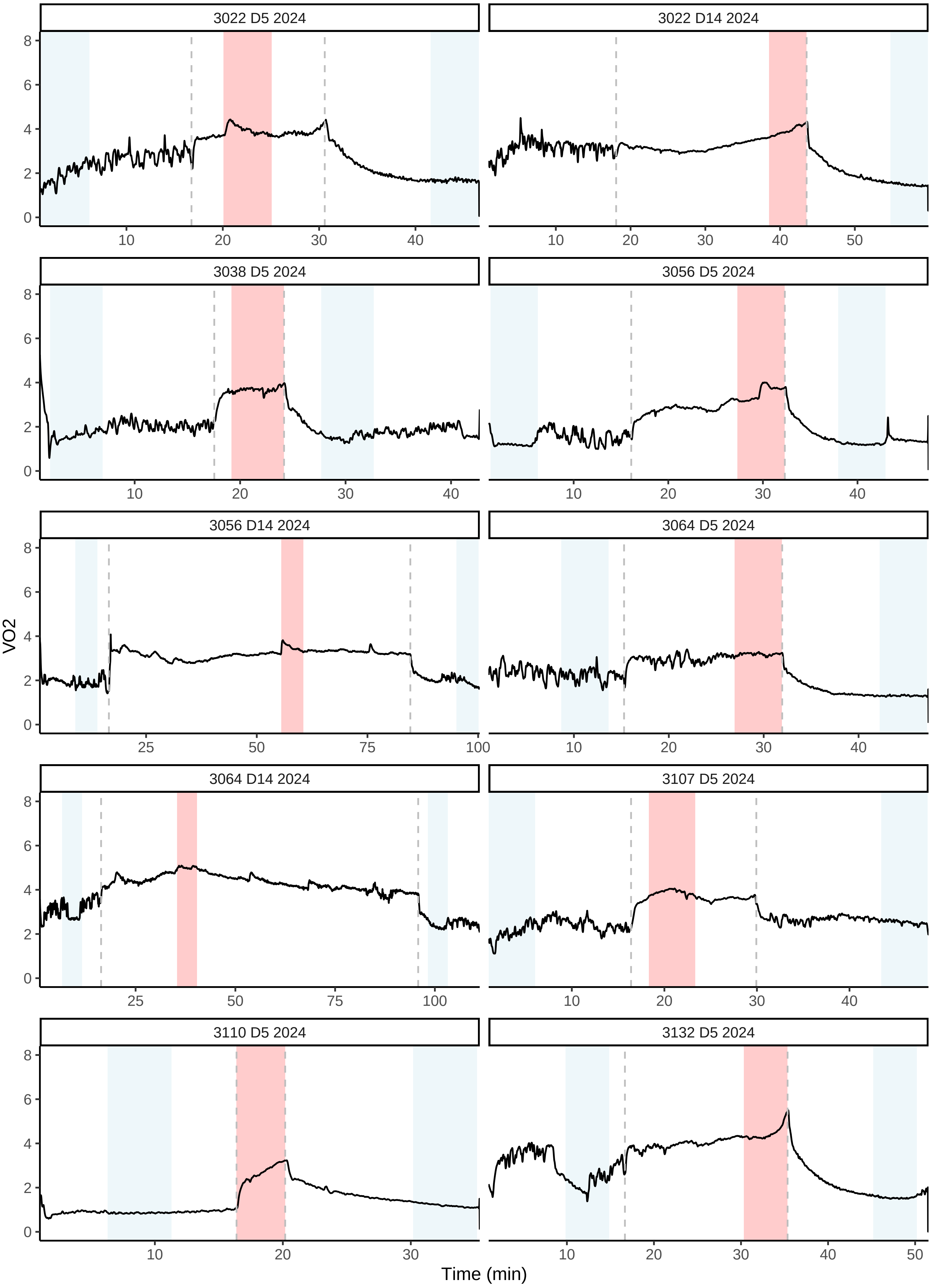

Journal of Experimental Biology • Supplementary information

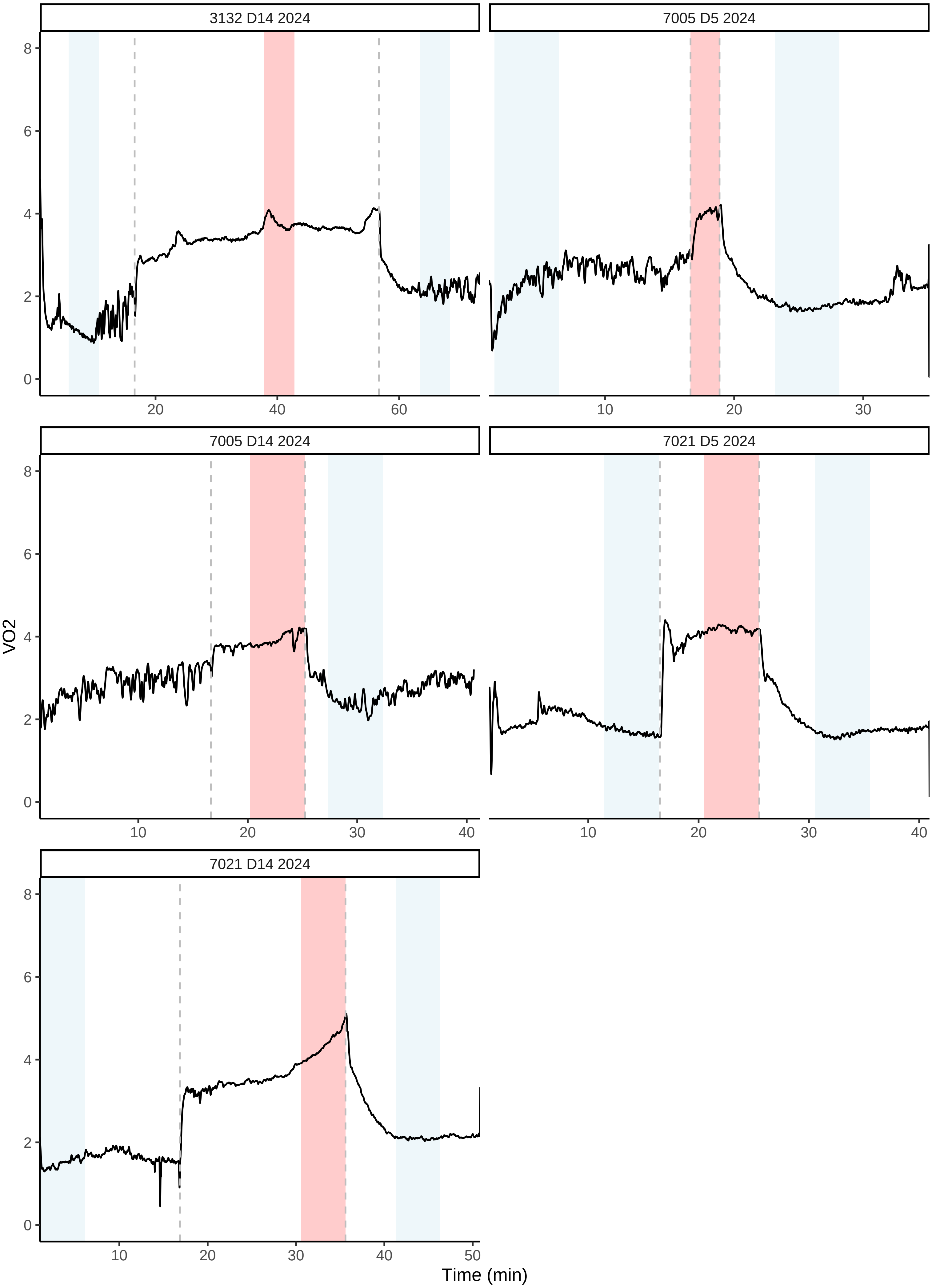

Fig. S2. Individual runs of all individuals with 5-minute selections of pre- and post-exercise metabolic rate marked in blue and a 5-minute selection of the maximum metabolic rate (MMR) marked in red. Time in minutes is shown on the x-axis and VO_2 (ml min^{-1}) is shown on the y-axis. The period when exercise was induced in a hop-flutter wheel is demarcated by dashed gray lines. Plots are labeled with the nest box ID, nestling age (days) and year.

Table S1. Results of linear models (LM) of the effects of a brood size manipulation on nestling blue tit wing length (mm) and tarsus length (mm) measured 9 days after, when they were 14 days old. Estimated marginal means of factors and slopes of co-variates (estimate), standard error (se), F-statistic and levels of significance are shown ($p < 0.05$ in bold and $p < 0.1$ in italics).

**Nestling wing length (mm)**					
Brood size			1, 69	3.22	*0.077*
Control	42.76	0.27			
Enlarged	42.04	0.28			
Parent age			1, 69	0.79	0.38
2CY	42.06	0.26			
3CY+	42.74	0.30			
Hatch date (April day)	0.065	0.050	1, 69	1.67	0.20
Year			1, 69	0.011	0.92
2023	42.38	0.28			
2024	42.42	0.29			
Brood size x parent age			1, 69	0.24	0.63
**Nestling tarsus length (mm)**					
Brood size			1, 69	2.01	0.16
Control	18.88	0.059			
Enlarged	18.78	0.062			
Parent age			1, 69	0.72	0.40
2CY	18.75	0.058			
3CY+	18.91	0.067			
Hatch date (April day)	-0.010	0.011	1, 69	0.82	0.37
Year			1, 69	3.03	0.086
2023	18.91	0.062			
2024	18.75	0.064			
Brood size x parent age			1, 69	0.56	0.46

Journal of Experimental Biology • Supplementary information
